# The Era of Precision Medicine: Advancing Treatment Paradigms for Small Cell Lung Cancer

**DOI:** 10.3390/cancers17111847

**Published:** 2025-05-31

**Authors:** Derek A. Corica, Scott D. Bell, Lei Zhao, Nicholas J. Lawler, McKade A. Poirier, Peyton J. Miller, Mark R. Wakefield, Yujiang Fang

**Affiliations:** 1 Department of Microbiology, Immunology & Pathology, Des Moines University, West Des Moines, IA 50266, USA; derek.a.corica@dmu.edu (D.A.C.); scott.d.bell@dmu.edu (S.D.B.); nicholas.j.lawler@dmu.edu (N.J.L.); mckade.poirier@dmu.edu (M.A.P.); peyton.j.miller@dmu.edu (P.J.M.); 2 The Department of Respiratory Medicine, the 2nd People’s Hospital of Hefei and Hefei Hospital Affiliated to Anhui Medical University, Hefei 230002, China; 19955873766@163.com; 3 Department of Surgery, University of Missouri School of Medicine, Columbia, MO 65212, USA; wakefieldmr@health.missouri.edu; 4 Ellis Fischel Cancer Center, University of Missouri School of Medicine, Columbia, MO 65212, USA

**Keywords:** small cell lung cancer, treatment

## Abstract

Small cell lung cancer (SCLC) remains a challenge prognostically. A clinically silent early stage and predilection for early metastasis leads to over half of patients presenting with metastatic disease at the time of diagnosis. Akin to many other cancers, once SCLC metastasizes, current therapies begin to lose their effectiveness. The future of SCLC rests in innovative treatments aimed at improving patient outcomes. Chemotherapy and radiation remain the backbone treatment for SCLC. Most patients diagnosed with SCLC begin treatment with combination chemotherapy consisting of a platinum analog and topoisomerase inhibitor with or without concurrent radiation. Disease progression or recurrence warrants new treatment approaches. New chemotherapy combinations and advances in radiation precision offer patients novel approaches using the same backbone of treatment used in many other cancers. The introduction of newer therapeutic approaches, such as immune checkpoint inhibitors, small molecule targeted therapies, bispecific antibodies, and antibody–drug conjugates offer a bright future for patients with SCLC who fail first-line therapy. This review will focus on advancing treatment paradigms for SCLC in the era of precision medicine. Such a study might be helpful for pulmonologists and oncologists to manage precisely patients with SCLC.

## Simple Summary

Small cell lung cancer is a challenging diagnosis for patients and physicians due to its high mortality rate. One reason small cell lung cancer is particularly deadly is its tendency to spread quickly throughout the body and its resistance to many existing treatment options. Since current treatment options are inadequate, it is necessary to develop new and improved treatment options to better manage small cell lung cancer. New treatment options, such as immunotherapy, antibody/medication combinations, and therapies aimed at unique cells, along with advances in traditional chemotherapy and radiation, have revolutionized the treatment of small cell lung cancer. This paper aims to explore these new treatment options and highlight the recent advances that have been made.

## 1. Introduction

Despite years of innovation and research, lung cancer continues to be a strain on the modern healthcare system. As of 2024, lung cancer ranked third in incidence and first in mortality worldwide [1]. In the United States alone, 125,070 patients lost their lives due to lung cancer in the past year [2]. Lung cancer takes many different forms and is categorized into two major groups, small cell and non-small cell forms. Non-small cell lung cancers (NSCLCs) comprise the majority of lung cancer diagnoses and include adenocarcinoma, squamous cell carcinoma, and large cell carcinoma [3]. Small cell lung cancer (SCLC) is categorized differently due to its histological and behavioral differences from NSCLCs. SCLC is a tumor derived from neuroendocrine cells and is known for its ability to grow rapidly and spread to distant body sites early in the disease process. While also relevant to NSCLCs, most patients with SCLC have a history of smoking [4].

Patient presentation of SCLC is highly variable, making diagnosis difficult. SCLC may present with classic respiratory symptoms, such as dyspnea, cough, or hemoptysis [4,5]. However, many cases have a more ambiguous clinical picture. Due to its neuroendocrine origin, SCLC tumors may release hormones and signaling peptides that cause a variety of neurological and paraneoplastic syndromes. The most common paraneoplastic presentations include Cushing syndrome, Lambert–Eaton myasthenic syndrome, hyponatremia of malignancy, and encephalomyelitis [6,7]. While it is less common for these syndromes to be the first presenting sign, their relationship with SCLC necessitates keeping SCLC on the differential diagnosis when treating patients who present with these syndromes. Additionally, it is not uncommon for a patient’s initial presentation to be signs of metastatic disease [5]. Imaging of a patient with SCLC typically reveals a centrally located mass within the lung field and subsequent biopsy is typically performed to achieve the diagnosis [4,5].

SCLC is relatively uncommon compared to NSCLCs, making up approximately 15% of overall lung cancer diagnoses worldwide. One of the most notable features of SCLC is its poor prognosis [4,8]. This poor prognosis is largely due to SCLC’s tendency to metastasize early in the course of the disease. In fact, approximately 2/3 of patients with SCLC display metastasis at the time of diagnosis [9]. Once SCLC metastasizes, the odds of survival become very poor. Currently, 5-year survival rates are 15–30% for local disease and below 7% for extensive-stage disease [10,11]. Surprisingly, SCLC is very responsive to initial treatment with chemotherapy and radiation, as 50–80% of patients experience an initial response [4]. What is particularly troubling is the high recurrence rate following an initial response to treatment. Moreover, recurrent SCLC develops increased resistance to existing therapies, making ongoing management especially challenging [4,11,12]. The high mutational burden of SCLC may help explain its resistance to treatment. Genomic studies have sought to uncover the key mutations driving its carcinogenesis. Among the most frequently identified alterations are inactivating mutations in *TP53* and *RB1*, observed in approximately 90% and 65% of SCLC cases, respectively [13]. Additionally, the activation of *EGFR* and members of the *MYC* proto-oncogene family has been reported in numerous cases of SCLC [14]. Interestingly, studies have also shown that mutations that result in decreased notch signaling are prominent as well, and may contribute to the poor cellular differentiation characteristics of SCLC [13,14]. Collectively, these genetic changes promote a more aggressive disease course and can diminish the effects of current therapeutic options.

The poor prognosis of SCLC highlights the urgent need for new, innovative treatments to improve patient outcomes. Many clinical trials are underway exploring novel treatments in hopes to improve long-term survival for patients with SCLC. The objective of this review is to highlight these recent advances and aid physicians in managing patients with this challenging disease.

## 2. Chemotherapy

Chemotherapy is the primary treatment modality for SCLC and plays a pivotal role in both limited-stage SCLC (LS-SCLC) and extensive-stage SCLC (ES-SCLC). Due to the rapid proliferation and inherent chemosensitivity of SCLC cells, systemic therapy is essential for controlling both local and metastatic disease.

### 2.1. First-Line Chemotherapy

For decades, the standard first-line chemotherapy regimen for SCLC has been a combination of a platinum-based DNA crosslinking agent (e.g., cisplatin or carboplatin) and a topoisomerase inhibitor (e.g., etoposide), with cisplatin and etoposide currently considered the standard of care [11,15,16,17]. These regimens have been extensively studied and are deemed safe for use as standalone treatments or in conjunction with systemic therapies such as radiation therapy, immunotherapy, or surgery [11]. Cisplatin and carboplatin are both widely utilized, but their use is associated with many adverse effects. Cisplatin is known for nephrotoxicity, ototoxicity, and gastrointestinal disturbances, though it has a lower incidence of myelosuppression compared to carboplatin [18,19]. Carboplatin, on the other hand, is favored in clinical practice due to its more favorable toxicity profile, particularly for patients with significant comorbidities or poor performance status [19]. Randomized trials and retrospective analyses in patients with SCLC have shown that cisplatin- and carboplatin-based regimens offer comparable efficacy. This finding is further supported by a meta-analysis of individual patient data from four randomized studies [19,20,21]. First-line chemotherapy for SCLC is typically administered over four to six cycles, with each cycle lasting three weeks [5]. In LS-SCLC, four cycles of chemotherapy are standard, while patients with ES-SCLC may receive up to six cycles depending on their response and tolerance to the initial regimen [5].

In recent years, the integration of immune checkpoint inhibitors into frontline therapy has marked a significant advancement in the treatment landscape of ES-SCLC. Landmark trials such as IMpower133 and CASPIAN demonstrated that the addition of immunotherapy to standard chemotherapy improves overall survival (OS) and progression-free survival (PFS), leading to the adoption of chemoimmunotherapy as the current standard of care for ES-SCLC [22,23].

IMpower133 (NCT02763579) demonstrated that the PD-L1 inhibitor atezolizumab combined with chemotherapy provided a survival benefit compared to chemotherapy plus placebo. Patients with chemotherapy-naïve ES-SCLC were treated with an induction phase of carboplatin/etoposide plus either atezolizumab or placebo for four twenty-one-day cycles. Following induction, patients continued on atezolizumab or placebo until disease progression or significant toxicity developed [22]. Patients in the atezolizumab group had a median OS of 12.9 months compared to 10.3 months in the placebo group [22]. Similar results were demonstrated in the CASPIAN (NCT03043872) study, when researchers investigated the PD-L1 inhibitor durvalumab with chemotherapy vs. chemotherapy alone in patients who had chemotherapy naïve ES-SCLC [23]. Patients underwent an induction period of chemotherapy plus either durvalumab alone, durvalumab with tremelimumab, or placebo for four cycles followed by maintenance therapy. The addition of durvalumab to chemotherapy resulted in a median OS of 13 months compared to 10.3 months with chemotherapy alone [23]. The positive results from IMpower133 and CASPIAN resulted in the recommendation that chemotherapy and immunotherapy be used in combination as the first line in the treatment of SCLC.

### 2.2. Maintenance Therapy

Maintenance therapy is generally not recommended for SCLC due to limited evidence supporting its benefit [24]. Instead, patients are monitored for disease progression and treated with second-line therapies upon relapse. Chemotherapy typically concludes after the planned cycles unless further disease progression is noticed.

### 2.3. Second-Line Therapy and Beyond

SCLC, while demonstrating significant sensitivity to initial chemotherapy, frequently experiences relapse or disease progression within a few months [25]. Despite initial responsiveness, the prognosis remains poor, with a 5-year survival rate below 10% [26]. For patients with relapsed SCLC receiving second-line therapy, the overall response rate (ORR) is approximately 51%, with a median PFS of 4.6 months [27]. Treatment options for relapsed SCLC are primarily determined by the duration of response to first-line therapy and patient-specific factors. For patients experiencing sensitive relapse (defined as relapse occurring more than 90 days after initial therapy), retreatment with the original chemotherapy regimen is often recommended [28,29]. In cases of resistant relapse (relapse within 90 days), sequential single-agent therapy is preferred [29].

Currently, two drugs are approved by the U.S. Food and Drug Administration (FDA) for the treatment of relapsed SCLC: topotecan and lurbinectedin [30,31]. Topotecan, a topoisomerase I inhibitor, interferes with DNA replication and transcription, ultimately leading to cancer cell death [32]. It is administered in cycles of five consecutive days every three weeks and is available in both intravenous and oral formulations. The intravenous form is often preferred for patients requiring the close monitoring of side effects, while the oral formulation offers a more convenient option for those who can tolerate outpatient therapy [28]. However, the use of topotecan is often limited by its side effect profile, which includes significant hematologic toxicities such as anemia, neutropenia, and thrombocytopenia. These toxicities necessitate close patient monitoring and supportive care measures during treatment [30].

Lurbinectedin is a newer agent with a distinct mechanism of action. It inhibits transcriptional activity in tumor cells and affects the tumor microenvironment by reducing the recruitment of inflammatory monocytes, thereby decreasing pro-tumorigenic signaling [33]. Lurbinectedin is administered as a single intravenous infusion every three weeks, offering both convenience and a more manageable toxicity profile compared to topotecan [31,33]. Common side effects include myelosuppression, fatigue, and gastrointestinal symptoms such as nausea, though these are generally less severe than those associated with topotecan [33]. Its approval was based on promising results from clinical trials, which demonstrated significant antitumor activity and durability of response in relapsed SCLC [31]. Despite the availability of topotecan and lurbinectedin, both agents have limitations in efficacy and tolerability, highlighting the need for continued research into novel therapies and combination regimens. Given these challenges, enrollment in clinical trials is strongly encouraged for all eligible patients [29].

## 3. Radiation Therapy

Radiation therapy (RT) is a critical component to the multimodal treatment approach to SCLC, with its application varying based on the disease stage and clinical circumstances.

### 3.1. LS-SCLC

Upon the diagnosis of LS-SCLC, the standard treatment for medically fit patients is concurrent thoracic radiotherapy and chemotherapy (radiochemotherapy) [17]. Evidence from two meta-analyses demonstrates that this combined modality approach significantly improves the 2-year survival rate compared to RT alone [34,35]. Most clinical guidelines recommend initiating thoracic radiotherapy early, ideally during the first or second cycle of cisplatin–etoposide chemotherapy [36,37]. Importantly, the interval between therapy initiation and the completion of RT serves as a key prognostic factor in LS-SCLC, with a prolonged interval correlating to a decrease in overall survival of approximately 1.9% per week [17,37,38]. While early concurrent radiochemotherapy is effective, it may not be appropriate for all patients. Individuals with large tumor burdens or poor performance status are at a higher risk of experiencing treatment-related toxicities with early RT. In such cases, deferring the initiation of RT or employing a sequential treatment approach is the preferred strategy [17].

The optimal dose and regimen for thoracic RT in LS-SCLC remains a subject of ongoing debate. Both once-daily and twice-daily radiation schedules have been evaluated in combination with etoposide and cisplatin chemotherapy. A randomized trial by Turrisi et al. (1999) demonstrated a modest survival advantage for twice-daily radiation over 3 weeks compared to once-daily radiation of 45 Gy over 5 weeks, with 5-year survival rates of 26% versus 16%, respectively. However, the twice-daily regimen was associated with a higher incidence of esophagitis [39].

The phase III CONVERT trial (NCT00433563) compared twice-daily RT (45 Gy in 30 fractions over 19 days) with once-daily RT (66 Gy in 33 fractions over 45 days), both starting on day 22 of cisplatin–etoposide chemotherapy. The primary endpoint, OS, was defined as the time from treatment initiation to death from any cause. Median OS was 30 months in the twice-daily group and 25 months in the once-daily group, with two-year OS rates of 56% and 51%, respectively, reflecting an absolute difference of 5.3% [40]. Although most toxicities were comparable between the groups, twice-daily radiation was associated with significantly higher rates of grade 4 neutropenia. Notably, unlike the earlier findings by Turrisi et al., the CONVERT trial did not observe significant differences in rates of esophagitis [39,40]. Despite its modest survival benefit, twice-daily RT has not been widely adopted [17,40]. Once-daily radiation to doses exceeding 60 Gy is feasible and commonly used, though its clinical advantages remain to be validated in phase III trials [40].

### 3.2. ES-SCLC

RT plays a critical role in managing metastatic disease and complications in patients with ES-SCLC, though palliative chemotherapy remains the standard treatment [5]. The prognosis for ES-SCLC is poor, with a median overall survival of 8 to 13 months [17,41]. For patients who respond to chemotherapy, thoracic radiation therapy (TRT) has shown additional benefits. The CREST trial (NTR1527) evaluated the impact of adding TRT (30 Gy in 10 fractions) to prophylactic cranial irradiation (PCI) in 498 patients with ES-SCLC. While the primary endpoint of 1-year OS showed no significant difference, secondary analyses revealed an improved 2-year OS of 13% vs. 3% in the controls, and 6-month PFS of 24% with TRT vs. 7% in the controls without TRT [42]. Intrathoracic recurrences were significantly reduced in the TRT group, and the treatment was well tolerated, supporting its use in selected patients who respond to chemotherapy [17,42].

Furthermore, PCI alone is another treatment option for patients who respond to chemotherapy. A randomized phase III trial (NCT00016211) involving 286 patients found that PCI significantly reduced the 1-year risk of brain metastases and improved both median disease-free survival and overall survival, with a higher 1-year survival rate in the PCI group [43]. However, the role of PCI in ES-SCLC has become increasingly controversial, particularly in the era of routine MRI surveillance. Subsequent studies, including a large Japanese phase III trial, have challenged the survival benefit of PCI, citing concerns over neurotoxicity and limited benefit in the context of regular imaging [44]. As a result, the use of PCI should be individualized, taking into account patient preferences, comorbidities, and access to MRI surveillance. In selected patients, particularly where regular brain imaging is feasible, MRI surveillance may be a reasonable alternative to PCI. While TRT is generally recommended for patients with intrathoracic disease who respond to chemotherapy, the decision to use PCI should be made on a case-by-case basis, reflecting the evolving evidence and ongoing debate regarding its overall benefit in extensive-stage SCLC [42,45].

### 3.3. Emerging Modalities in RT

The future of RT in SCLC lies in the advancement of techniques aimed at improving precision, efficacy, and patient outcomes. Emerging strategies include integrating intensity-modulated radiotherapy (IMRT), volumetric-modulated arc therapy (VMAT), stereotactic body radiotherapy (SBRT), and adaptive radiation therapy (ART), all of which aim to target tumors with greater accuracy while minimizing toxicity.

IMRT is an innovative technique that optimizes radiation dose distribution in three dimensions, delivering precise doses to tumors while sparing surrounding healthy tissues [46]. The ability to modulate beam intensity enables the delivery of higher radiation doses to tumors, potentially improving local control and overall outcomes [47]. While data on the use of IMRT in SCLC are limited, it may be beneficial for targeting thoracic tumors near critical structures, such as the heart and esophagus, reducing the risk of treatment-related toxicities like esophagitis and pneumonitis [48].

VMAT, a type of IMRT, continuously delivers radiation as the treatment machine rotates around the patient, resulting in shorter treatment times and highly conformal dose distributions [49]. Both VMAT and IMRT are commonly used in lung cancer treatment, with most published data focusing on planning and feasibility studies. However, emerging clinical outcomes are available for various tumor sites, including lung cancers [50].

SBRT delivers high-dose radiation with exceptional precision in a limited number of fractions, making it ideal for treating localized or oligometastatic disease [51]. In SCLC, SBRT is primarily used for brain or limited thoracic metastases, offering effective disease control in cases where conventional RT may be less practical. Its precision minimizes radiation exposure to surrounding healthy tissues, making it a favorable option for patients with comorbidities or reduced treatment tolerance. While SBRT is the standard treatment for medically inoperable patients with early-stage NSCLC [46], its application in SCLC should follow established NSCLC treatment guidelines [52].

ART uses real-time imaging to adjust treatment plans based on anatomical changes, such as tumor shrinkage or shifts in organ position during treatment [53]. ART ensures that radiation is consistently delivered to the intended target while minimizing exposure to the surrounding healthy tissues. Additionally, it allows for the dynamic redesign of radiation treatment plans to account for changes in tumor size and location [54]. While studies on ART for lung cancer management have primarily focused on NSCLC, there are limited data addressing its use in LS-SCLC [53]. However, a few studies, case reports, and research involving both NSCLC and SCLC patients suggest that ART may improve treatment outcomes in SCLC by reducing normal tissue exposure, potentially allowing for dose escalation to optimize tumor control, especially in patients undergoing concurrent chemoradiation [53].

As the field of RT evolves, new modalities such as IMRT, VMAT, SBRT, and ART offer promising advancements for the treatment of SCLC. These techniques provide greater precision in targeting tumors, which may improve local control while reducing toxicity to surrounding healthy tissue. However, further research and clinical studies are needed to fully understand their benefits, limitations, and optimal use in the context of SCLC. Incorporating these advanced RT modalities into treatment protocols may enhance the management of SCLC, offering patients improved outcomes and a better quality of life.

## 4. Immunotherapy

While the immune system is highly effective at combating infections, its ability to target cancer is hindered by several challenges. Activation of the immune system requires the recognition of antigens that are unique to pathogens. Once a pathogen specific antigen is recognized, the immune system can mount a robust response to eliminate the invader. In contrast, cancer cells originate from the body’s own tissues and generally express the same antigens and proteins as normal, healthy cells. Without distinct markers to signal that these cells are abnormal or foreign, the immune system often fails to recognize cancer as a threat, allowing it to evade immune detection and destruction [55]. Moreover, mutations to the cancer cell genome provide additional methods for evading detection by the immune system. This constellation of mutations can lead to an immunosuppressive tumor microenvironment that dampens the immune response taking place. By reducing cytokine production, impairing major histocompatibility complex (MHC) expression, and suppressing T-cell activation, cancer cells can effectively escape immune surveillance. As a result, the immune system becomes functionally “blind” to their presence, allowing the tumor to grow and spread unchecked [56,57]. Despite the many mechanisms cancer cells use to evade the immune system, substantial evidence indicates that the immune system can, under the right conditions, mount an effective response against cancer. For example, solid tumors with higher levels of T-cell infiltration are often associated with improved prognosis [58]. Observations like these, and many more, have sparked a growing interest in using the immune system to fight cancer, which has effectively given rise to the field of immunotherapy. Immunotherapy aims to increase effective immune responses against cancer cells by modulating the tumor microenvironment to a more “pro-immune” phenotype. Today, immunotherapy is deployed in many types of cancer. Approaches such as immune checkpoint inhibitors (ICIs), tumor vaccines, and cell mediated therapies have all provided durable responses when used to treat many cancers [59,60,61,62].

### 4.1. Immune Checkpoint Inhibitors

The discovery of immune checkpoint proteins represented a major leap in the field of immunotherapy. Checkpoint proteins, located on immune cells, are potent suppressors of the immune system that prevent overzealous immune responses [63]. While these proteins serve an important biological role in the prevention of immune mediated cellular damage and autoimmunity, certain mutations in tumors allow for the exploitation of these proteins as a means of immunosuppression [57]. To date, the most studied immune checkpoint proteins are cytotoxic T-lymphocyte antigen-4 (CTLA-4), programmed death 1 (PD-1), and its ligands PD-L1 and PD-L2 [64,65,66,67].

The interaction between CTLA-4 and co-stimulatory molecules CD80 and CD86 represents one of the key immunosuppressive mechanisms in the body. When activated, CTLA-4 binds to CD80 and CD86 on antigen presenting cells (APCs), resulting in decreased T-cell proliferation and activity [64,68,69,70]. Research into CTLA-4’s immunosuppressive role has demonstrated that CTLA-4 deficient mice develop unregulated T-cell proliferation and autoimmune disorders [71]. Conversely, studies have shown that anti-CTLA-4 therapy enhances T-cell activity and stimulates robust immune responses to tumor cells [72,73,74]. Considering these findings, inhibition of the CTLA-4 axis has sparked research into its use for the treatment of SCLC. Arriola et al. investigated the addition of ipilimumab, an anti CTLA-4 antibody in combination with carboplatin and etoposide in patients with chemotherapy naive ES-SCLC. Patients experienced a median immune related progression free survival (irPFS) of 7.3 months with 84.8% experiencing an objective response [75]. Additionally, Reck et al. demonstrated that when combined with paclitaxel/carboplatin, ipilimumab provides a survival benefit for patients with ES-SCLC vs. chemotherapy alone. The median OS was 12.9 months for ipilimumab plus chemotherapy vs. 9.9 months for chemotherapy alone [76]. These studies demonstrate that anti CTLA-4 therapies are effective additions to standard chemotherapeutic regimens in the treatment of SCLC. However, additional studies in patients with ES-SCLC investigating ipilimumab combined with chemotherapy failed to show a significant difference in OS, 11.0 months vs. 10.9 months, respectively [77]. These mixed outcomes highlight the potential of anti-CTLA-4 therapies; however, further studies are needed to clarify their role in the treatment of SCLC.

The PD-1/PDL1 axis represents another important mechanism to regulate the immune system. Similar to CTLA-4, activation of the PD-1/PDL1 axis suppresses immune system activity. This interaction plays a crucial role in preventing the development of autoimmune diseases and excessive immune responses to pathogens. PD-1 is a receptor expressed on T-cells, B-cells, and NK cells, while its ligands, PD-L1 and PD-L2, are expressed on both APC and non-immune cells [78]. When PD1 binds to its ligands PD-L1 or PD-L2, it triggers inhibitory signaling pathways that promote T-cell apoptosis, thereby dampening immune responses [65,79]. Cancer cells exploit this mechanism by upregulating PD-L1 and PD-L2 expression, effectively evading destruction by cytotoxic T cells [78]. Akin to the CTLA-4 axis, the inhibition of the PD-1/PD-L1 axis removes the breaks from the immune system, allowing for increased cancer destruction. Targeting this axis has become a success story in cancer immunotherapy, exhibiting durable responses for numerous advanced stage cancers, which has led to further research into the use of PD-1/PD-L1 inhibitors to treat aggressive cancers like SCLC [65,66,67].

The ADRIATIC trial (NCT03703297) is considered a landmark study demonstrating the efficacy of PD-1/PD-L1 inhibition on survival outcomes in SCLC. Patients with LS-SCLC that showed no disease progression after standard chemoradiotherapy were assigned in a 1:1:1 manner to receive the PD-L1 inhibitor durvalumab plus tremelimumab, durvalumab alone, or placebo. This was later amended to remove the durvalumab–tremelimumab group after data suggested survival benefits with durvalumab alone [80]. This amendment led to a new 1:1 assignment into the durvalumab or placebo groups. Durvalumab showed significant effects vs. placebo, with a median OS of 55.5 months vs. 33.4 months and PFS of 16.6 months vs. 9.2 months, respectively [80]. Additionally, a three-year interim analysis revealed a three-year OS of 56.5% in the durvalumab group vs. 47.6% in the placebo group [80]. These results are positive, as they outpace the current five-year median OS for patients being treated with standard chemoradiotherapy. As an adjuvant treatment, durvalumab was able to extend survival in patients who failed conventional chemoradiotherapy, asserting the use of PD-L1 inhibitors should be used in treatment regimens for SCLC.

Another anti-PD-1 antibody, nivolumab, was studied by Ready et al. as a monotherapy for patients with recurrent SCLC who had previously undergone chemotherapy. The study reported an objective response rate of 11.9%, with a median duration of response lasting 17.9 months at follow-up [81]. Additionally, pembrolizumab, a PD-L1 inhibitor, demonstrated a 19.3% objective response rate as a monotherapy in treatment-refractory SCLC, with two patients reporting complete responses [82]. Together, these studies highlight the potential effectiveness of targeting the PD-1/PD-L1 axis in patients with treatment-resistant SCLC. While the impact of these studies is notable, both pembrolizumab and nivolumab are no longer approved as monotherapy for the treatment of small cell lung cancer. Both approvals were revoked in 2021 by the FDA due to failed improvement in clinical outcomes [83].

While PD-1/PD-L1 inhibition as monotherapy has shown promise, the greatest treatment benefits have been observed when these inhibitors are combined with chemotherapy. For example, first-line treatment with pembrolizumab plus etoposide and platinum (EP) demonstrated a PFS advantage over chemotherapy alone in patients with untreated extensive-stage SCLC. Specifically, 13.6% of patients receiving pembrolizumab maintained PFS at one year, compared to just 3.1% in the control group [84]. A summary of these studies is provided in Table 1. Overall, these findings support the use of PD-1/PD-L1 axis inhibition as a valuable treatment option for patients with SCLC.

While these treatment benefits are promising, like chemotherapy, immunotherapy can be influenced by the tumor’s mutational status. SCLC is characterized by a notably high tumor mutational burden (TMB), partly driven by the frequent concurrent deletion of tumor suppressor genes *TP53* and *RB1* [13]. Deletions of *RB1* occur in over two-thirds of SCLC patients and significantly impact the success of immune checkpoint inhibitor therapy. For instance, patients with *RB1* mutations had a median OS of 5 months, compared to 23.1 months for those with wild-type *RB1* receiving ICIs [85]. Conversely, several studies have found that a higher TMB is associated with better treatment outcomes [86,87]. Although the mutational landscape of SCLC is still in its infancy, advancements in understanding will enhance the ability to predict which patients are likely to benefit from ICIs.

The safety profile of ICIs remains a significant obstacle in their clinical use for SCLC. Most patients treated with ICIs experience adverse events (AEs), which occur slightly more frequently than in placebo groups [23,84]. The side effects are generally similar between anti-CTLA-4 and anti-PD-1/PD-L1 therapies and commonly include anemia, thrombocytopenia, alopecia, nausea, and gastrointestinal symptoms such as constipation or diarrhea [23,75,76,77,84].

Despite these challenges, ICIs hold great promise for SCLC treatment by “releasing the brakes” on the immune system through inhibition of immunosuppressive CTLA-4 and PD-1/PD-L1 pathways. This reinvigoration of the immune response against cancer cells has translated into durable survival benefits in both LS-SCLC and ES-SCLC disease, whether used alone or in combination with frontline therapies. However, side effects and tumor mutational burden limit efficacy in certain patients.

The future success of ICIs depends on focused research addressing these key issues. Mitigating side effects could prevent premature treatment discontinuation and allow patients to complete full therapy courses. Additionally, deeper insights into TMB and specific genetic mutations influencing treatment response will enable more precise patient selection and tailored therapeutic approaches, ultimately improving outcomes for those battling SCLC.

### 4.2. Oncolytic Viruses

**Figure 1:**
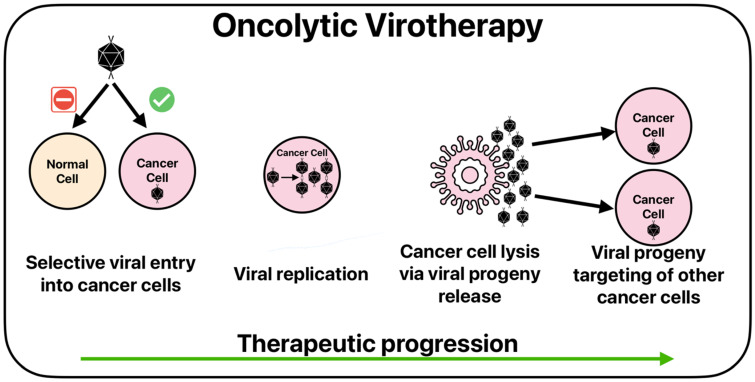
The basic function of OV.

Oncolytic virotherapy (OV) is a novel field of study that investigates the use of viruses to target cancer. OV utilizes both natural and engineered viruses specifically targeted at cancer cells while sparing normal cells. The process of viral infiltration, replication, and release of progeny leads to cancer cell death, as illustrated in Figure 1 [88,89]. Beyond direct tumor destruction, OV also stimulates a robust antitumor immune response. The lysis of cancer cells releases tumor associated antigens (TAAs) and pathogen-associated molecular pattern molecules (PAMPs) which can be picked up by APCs and presented to T-cells, activating the immune system against tumor cells [90]. Immune activation can also be assisted by genetic engineering to express proteins that activate the immune system [91]. OV offers a safe and specific method of treatment for patients who are unresponsive to other treatment methods. Currently, there are several OVs approved for cancer treatment, including talimogene laherparepvec (T-Vec) for advanced melanoma and teserpaturev for malignant glioma, among others [92,93].

To date, most studies investigating OV for SCLC have been conducted on mouse models or in vitro. Li et al. demonstrated that the recombinant adenovirus Ad-Apoptin-hTERTp-E1a (Ad-Vt), combined with etoposide, inhibited SCLC proliferation in mice by activating mitochondrial apoptotic pathways linked to viral infection. This treatment also extended survival times in the animal models [94]. OV outcomes on SCLC are similar, regardless of route of administration. Direct delivery of a modified oncolytic myxoma virus (MYXV) in both murine and human SCLC cells resulted in widespread tumor necrosis [95]. Additionally, the systemic administration of Seneca Valley Virus-001 (SVV-001) in mice with xenografted SCLC resulted in a complete reduction of tumor mass in all 60 mice at doses above 1 × 10^8^ virion particles per kg [96]. Human trials, however, have shown mixed results. SVV-001 was tested in patients with SCLC and other mixed neuroendocrine tumors, but none achieved an objective response per RECIST criteria; nonetheless, one patient showed slowed disease progression following viral infusion [97]. Despite the lack of consistent clinical efficacy, these studies consistently demonstrated strong viral tropism for SCLC cells with minimal impact on normal cells [94,95,96,97]. While OV has shown promise in treating other cancers, its application in SCLC remains under investigation. Currently, no oncolytic virus therapies have been approved for SCLC, and human trial results have yet to provide conclusive evidence of benefit.

## 5. Targeted Therapy

Targeted drug therapies in the treatment of SCLC are being used more often due to an improved understanding of the disease’s molecular biology and genomic composition [98,99]. Though chemotherapy is still the cornerstone of SCLC treatment, targeted therapies are currently being used in clinical trials and even in some late-stage treatment settings for SCLC refractory to chemotherapy treatment. These therapies aim to provide a tailored approach to the treatment of SCLC, rather than treating it as a monolithic disease [100,101]. Among the targeted therapies being researched in clinical practice are poly ADP–ribose polymerase (PARP) inhibitors, delta-like ligand 3 (DLL3) inhibitors, and B-cell lymphoma 2 (BCL-2) inhibitors.

**Figure 2:**
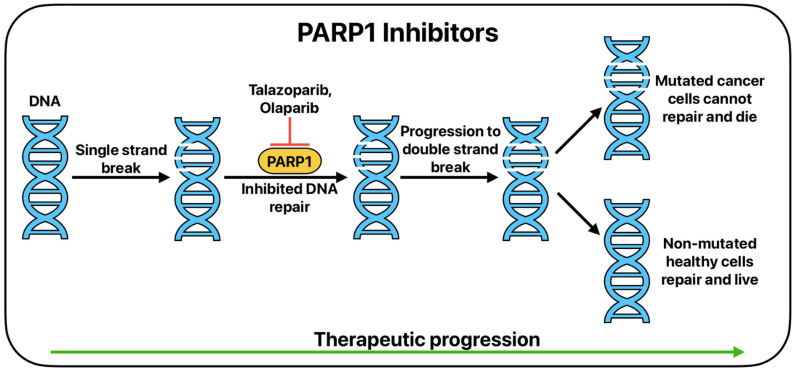
The basic function of PARP1 inhibition in cancer therapy.

PARP1 is the major protein in the PARP protein family that activates base excision after the occurrence of single-strand breaks (Figure 2) [102]. PARP1 is capable of recruiting proteins and DNA repair factors to the site of DNA damage to initiate repair [103]. This protein typically attaches ADP-ribose groups to acceptor proteins to enhance DNA repair. Talazoparib is a PARP inhibitor that is FDA approved for use in breast and prostate cancer pathologies [104,105]. Olaparib, a PARP inhibitor, is currently FDA-approved for use in treatment of partial or completely platinum-based chemotherapy responsive ovarian, fallopian tube, and peritoneal cancer [106]. Niraparib is another PARP inhibitor that is currently FDA-approved for use in both ovarian and prostate cancer [107]. Parp1 has shown irregular expression patterns in SCLC, thus making it a prime target for drug interaction and targeted treatment [103,108,109,110,111]. Schlafen family member 11 (SLFN11) DNA/RNA helicase has been identified as a biomarker that when upregulated in SCLC, correlates with increased cancer destruction when PARP inhibitor therapy is introduced [112]. Further, it has been shown that expression of O6-methylguanine-DNA methyltransferase (MGMT), an enzyme that repairs DNA after alkylating damage, is a predictor of PARP inhibitor–temozolomide combination therapy success. In MGMT-positive cancer cell lines, there is an increased response to PARP inhibitor–temozolomide combination therapy [113]. Targeted PARP inhibitor therapy presents as a promising treatment route for SCLC, with even more potential when used in combination therapy in the future (Table 2).

BCL-2 is an oncoprotein that is overexpressed in many cancers, including SCLC [117,118,119]. BCL-2 is an anti-apoptotic protein that is expressed on the surface of the outer mitochondrial membrane that inhibits the activation of pro-apoptotic proteins, such as BAX and BAK, which leads to the inhibition of caspase activity [120]. Further, BCL-2 functions to maintain the permeability of the outer mitochondrial membrane to ensure that no irreversible cellular damage occurs. Cells that overexpress BCL-2 are capable of inhibiting apoptosis and are sturdier than non-expressing cells. When BCL-2 is inhibited, cellular destruction can occur; thus, BCL-2 presents as a promising target in the treatment of SCLC. A phase one study investigating navitoclax, BCL-2 family inhibitor, found that the treatment of grade one or two SCLC resulted in a stable disease, with one patient having a partial response time greater than two years. Tahir et al. found that ABT-737, a BCL-2 inhibitor, was effective against in vitro SCLC cell lines that expressed BCL-2 [121]. Further, Shoemaker et al. found that ABT-263, another BCL-2 inhibitor, caused tumor regression in SCLC xenografts, further highlighting the potential therapeutic application of this inhibitor family in clinical practice [122].

## 6. Bispecific Antibodies

Bispecific antibodies (bsAbs) are engineered molecules that have been designed to bind two distinct antigens simultaneously. This action facilitates a targeted therapeutic strategy in cancer treatment. Bispecific antibodies were first investigated in 1961 by Nisonhoff and Rivers in animal models [123]. In 1983, the first bispecific antibody was developed by Milstein and Cuello with a large promise for future medical use [124]. Since then, many bsAbs have been developed, evaluated, and utilized for cancer treatment. They have shown precision in targeting and enhancement of binding compared to traditional cancer drugs. These bsAbs offer opportunities for more efficacious treatment of cancers like SCLC, where some immunotherapy treatments have not been particularly promising [125]. A key target of bsAbs in treatment of SCLC is DLL3 on tumor cells. When using bsAbs to target DLL3 with one arm and CD3 on T-cells more efficacious treatment of SCLC may be achieved and has offered new avenues for this disease with previously limited options.

### 6.1. Bispecific Antibody Structure

BsAbs are designed to have two distinct target arms with binding domains for the targeting of two distinct antigens. This contrasts with naturally occurring antibodies which have two target arms that both bind the same antigen. BsAbs have two general categories that distinguish them: IgG-like which contains an Fc region and non-IgG-like subtypes without an Fc region.

### 6.2. Use in Small Cell Lung Cancer

SCLC remains a challenging disease to treat, with many immunotherapy options being limited by efficacy and lack of identifiable markers. This has led to a poor prognosis for patients with SCLC and has encouraged the development of new immunotherapy modalities such as bsAbs.

### 6.3. DLL3-Targeted

DLL3 is a common target of bsAbs due to its expression on the surface of over 80% of SCLC tumor cells. Tarlatamab is a bispecific T-cell engager (BITE) antibody which binds DLL3 and CD3 on T-cells. This aids immune system targeting and subsequent T-cell dependent killing of tumors. In 2017, a phase I study of tarlatamab (AMG757) was launched to evaluate its safety, efficacy, and pharmacokinetic data [126]. Sixty-four patients were enrolled in the study at 10 varying dosages with anti-tumor activity seen across the dose ranges. AMG757 revealed rapid and durable responses for patients with SCLC and safety with doses up to 100 mg. A 2022 study evaluated tarlatamab in relapsed/refractory SCLC [127]. In the study, 107 patients were treated and 97 experienced an AE. The most common AE was cytokine release syndrome, which occurred in 52% of patients. However, efficacy data were encouraging with achievement of a 23.4% (95% CI: 15.7–32.5) objective response rate. Additionally, two patients were reported to have complete remissions and 23 with partial remissions. A disease control rate of 51.4% (95% CI: 41.5–61.2). The median PFS and median OS were 3.7 months (95% CI: 2.1–5.4) and 13.2 months (95% CI: 10.5 to not reached), respectively. These results are encouraging, offering a longer median survival for SCLC patients. Tarlatamab has solidified an efficacious use of bsAbs and a reliable safety profile for SCLC patients.

BI 764532 is another BITE antibody which also binds DLL3 and CD3. BI 764532 has demonstrated potent tumor cell lysis and T-cell infiltration of tumor tissue in some cases leading to complete tumor regression in SCLC patients [128]. An additional finding in this study by Hipp et al. was the upregulation of PD-L1 and LAG-3, which may suggest efficacy of BITE and immune checkpoint inhibitory combination therapy. A more recent study from 2022 containing tumor response rates from 24 SCLC patients demonstrated an objective response rate of 33% across all regiments of BI 764532 [129]. Similar to tarlatamab, a tolerable safety profile was achieved with the most common AE being cytokine release syndrome managed with supportive care. While promising efficacy with BI 764532 has been demonstrated, clinical trials (NCT04429087) are still underway assessing MTD.

Similar to AMG757 and BI 764532, PT 217 is a bispecific antibody that targets DLL3 with one of its arms. However, it is unique in that its second arm targets CD47 which is expressed by SCLC to prevent macrophage-mediated destruction of tumor cells. In a 2022 study, it was found to have antitumor activity against SCLC in mouse models through two main routes [130]. The first is by blockading the CD47-SIRPa interaction which normally stimulates macrophage phagocytosis of tumor cells. Second, it was found to deliver potent NK-cell-mediated cytotoxicity against tumor cells. In addition, it demonstrated a good safety profile similar to other DLL3 targeted bispecific antibodies. Overall, DLL3 targeted bispecific antibodies have shown promising efficacy in objective remission rate for SCLC patients.

### 6.4. LAG-3-Targeted

The lymphocyte activation gene-3 (LAG-3) encodes a type I transmembrane protein located on chromosome 12 with an extracellular, transmembrane, and intracellular region [131]. This gene has been investigated for targeting by bispecific antibodies due to its antitumor potential, specifically its intracellular domain which stimulates T-cell proliferation and cytolytic functions [132]. LAG-3 is not typically expressed on early T-cells, but its expression can be induced by CD4+ and CD8+ T-cells [133]. When examining T-cell expansion and LAG-3, there is a negative correlation found. This leads to a problem within tumors where T-cells are repetitively stimulated, leading to depletion and increased expression of LAG-3. Ultimately, this leads to a loss of sensitivity of T-cells and less antitumor activity [134]. Therefore, by targeting and inhibiting LAG-3, T-cell activity increases and promotes antitumor ability [135]. These findings have promoted an investigation of LAG-3 inhibition into cancer therapy using bispecific antibodies.

XmAb^®^22 841 is an anti-CTLA4-LAG-3 bispecific antibody that is designed to increase the T-cell response and expansion. It has been evaluated in a phase I study (NCT03849469) for dosage as monotherapy and in combination with pembrolizumab. While the main investigation for this study involves efficacy of the drug in prostate cancer and its metastases, its mechanism holds promise for other solid tumor cancers such as SCLC. The results derived from this study are still pending publication.

### 6.5. GD2-Targeted

GD2 is a glycolipid that partakes in cell-to-cell interactions, and is overexpressed in sarcomas, neuroblastomas, and SCLC [136]. When expressed in SCLC, GD2 can increase cancer cell proliferation, adhesion, invasion, and antiapoptotic qualities [137]. This aids tumor cells in immune evasion and makes GD2 a hopeful target of therapy.

Nivatrotamab (Hu3F8-BsAb) is a bispecific antibody that targets GD2 with one arm and CD3 with the other. One study, NCT04750239, has been conducted to evaluate the safety of Nivatrotamab. However, the study was terminated due to business priorities after three patients.

Bispecific antibodies mark a great advancement in therapy for SCLC and provide patients with more options for cancer with limited treatments and poor prognosis. Utilizing the dual targeting structure of bsAbs and a variety of immunological mechanisms, they have demonstrated promising efficacy and safety profiles in clinical trials. While demonstrating a tolerable safety profile, cytokine release syndrome was the most reported side effect across these bsAb trials. As of May 2024, the only FDA approved bsAb for SCLC is tarlatamab for ES-SCLC [138]. There continues to be an ongoing investigation with clinical trials on bsAbs targeting multiple immune mechanisms. Many of these trials are in their infancy, as most of them are undergoing or completing phase I. The progression of these trials is promising and may provide new effective methods for SCLC treatment. Another avenue of research for bsAbs use will be investigating their efficacy across different subtypes of SCLC. Most SCLC tumors express transcription factors ASCL1 (SCLC-A) and NEUROD1 (SCLC-N). DLL3 is expressed in these as well as other neuroendocrine SCLC subtypes, justifying therapies like tarlatamab and making it a great target for future research [139,140]. However, there are other subtypes of SCLC such as POU2F3 and YAP1-like tumors that lack investigation. Continuing investigation of immune targets such as DLL3, LAG-3, GD2, EFGR, and more will be important for future research to counteract resistance mechanisms of SCLC against bsAb therapy. Further research in this area will also help provide improved safety profiles and tailored therapy options for patients.

## 7. Antibody–Drug Conjugates

Antibody–drug conjugates (ADCs) have emerged as a targeted therapy for SCLC. Specific antibodies allow for delivery of chemotherapeutic agents directly to cancer cells by targeting specific tumor markers [141,142]. The targeted delivery of these agents reduces systemic and toxic side effects of cytotoxic drugs [143]. ADC treatments for SCLC are an emerging treatment modality, therefore most of the treatments discussed in this section are still in the early stages of research and development.

### 7.1. DLL3

The first ADCs that emerged to treat SCLC targeted DLL3 [141], the same marker discussed in the bsAbs section. DLL3 functions as a notch pathway inhibitor that is upregulated and expressed on the surface of SCLC tumor cells [142]. DLL3 promotes SCLC growth, proliferation, and migration of SCLC cells [143,144].

Rovalpituzumab tesirine (Rova-T) is an ADC that combines a humanized monoclonal antibody targeting DLL3 (SC16) with pyrrolobenzodiazepine (PBD) dimer toxin [143,145]. After binding to DLL3, the PBD dimer toxin is internalized by the SCLC cell resulting in DNA damage and apoptosis [145,146]. Initial phase I data for Rova-T were encouraging with a 36% 1-year survival as a third-line treatment in patients with high levels of DLL3 expression [147]. Treatment responses to Rova-T were not as promising in patients with low DLL3 expression [147]. Subsequent Rova-T phase II and III studies have been less encouraging and demonstrated significant adverse effects [143]. The TRINITY phase II study demonstrated modest antitumor activity with an overall ORR of 14.3% and median overall survival of 5.7 months in patients with high levels of DLL3 expression [148]. This study also found significant adverse effects associated with Rova-T including pleural effusion, thrombocytopenia, pericardial effusion, and photosensitivity reactions [148]. Additionally, the TAHOE trial, a phase III randomized controlled trial, found that Rova-T had a shorter median OS (6.4 months), PFS, and lower ORR when compared to topotecan (median OS 8.6 months) as a second-line therapy for DLL3-high SCLC patients [149]. The MERU trial, another phase III trial, also failed to demonstrate Rova-T survival benefit versus placebo [150]. Due to safety concerns and mild improvement in survival, Rova-T development has been discontinued [151].

### 7.2. Trophoblastic Cell Surface Antigen (TROP2)

Trophoblastic surface antigen 2 (TROP2) is a transmembrane glycoprotein that acts as a calcium signal transducer [152]. TROP2 expression is increased in many solid tumors including SCLC making it a viable target for ADCs [153,154]. Increased TROP2 expression promotes cell proliferation, survival, and invasion [152].

Sacituzumab govitecan (SG) is an antibody–drug conjugate directed at TROP2 that has recently emerged as a potential treatment for SCLC. Sacituzumab govitecan consists of a monoclonal antibody targeting TROP2 linked to the topoisomerase inhibitor SN-38 [143]. After binding TROP2, SN-38 is taken up by the cancer cell producing cytotoxic effects by inhibiting topoisomerase I [143]. A phase I/II study conducted by Baria et al. explored the efficacy of SG for various refractory metastatic epithelial cancers including SCLC. This study found an ORR of 17.7% in SCLC patients with a median overall survival of 7.1 months and median PFS of 3.7 months [154]. Preliminary results from a phase II trial (NCT03964727) exploring SG efficacy in patients with extensive stage SCLC found an ORR of 29%. Additionally, none of the patients in this trial had to discontinue treatment due to treatment-related adverse effects [155]. SG may also provide benefit when combined with platinum-based chemotherapies for SCLC [156].

### 7.3. B7-H3

B7-H3, also called CD276, is a B7 family protein that functions as a T-cell regulator that is overexpressed in many cancers including SCLC [156]. Increased expression of the B7 family proteins may contribute to tumor cell’s ability to evade the immune system [156,157]. B7-H3 has emerged as a potential ADC target.

DS-7300 is an antibody–drug conjugate targeting B7-H3 that has been studied for the treatment of SCLC [153]. DS-7300 is a B7-H3 targeted monoclonal antibody conjugated to the topoisomerase I inhibitor, deruxtecan. A phase I/II study (NCT04145622) exploring efficacy of DS-7300 on heavily pretreated patients with advanced SCLC was recently conducted [158]. Of the 21 patients included in the analysis of this study, 11 (52%) had an objective response, median PFS was 5.8 months, and median OS was 9.9 months as of January 31st 2023 [159]. Another phase II study (NCT05280470) exploring the efficacy of DS-7300 on end-stage SCLC interim analysis found that patients treated with the higher 12-mg/kg dose of DS-7300 had an ORR of 54.8%, median duration of response, PFS, and OS of 4.2, 5.5, and 11.8 months respectively [160,161]. Response was much better in the 12-mg/kg dose group when compared to the 8-mg/kg dose group [161]. A phase III study (NCT06203210) comparing DS-7300 to a treatment of physician’s choice for patients with relapsed SCLC is in the recruiting phase as this is being written [162]. The results for DS-7300 have been promising, but its development is still in the early stages.

## 8. Conclusions

SCLC remains a cancer associated with high mortality due to its early metastatic spread and high mutational burden. Over the years, first-line treatment for SCLC remains a combination chemotherapy with or without radiation due to SCLCs great susceptibility profile to these modalities. Many patients will show initial treatment gains. However, most will also experience relapse and recurrence of their disease. Despite these facts, the future of SCLC treatment remains bright due to the ever-expanding catalogue of studies investigating the efficacy of new and improved treatment methods. ICIs exploiting the CTLA-4 and PD1/PDL1 axes when combined with chemotherapy show impressive gains in both first- and second-line treatments and may one day become the gold standard of treatment for SCLC. Both bsAbs and ADCs take advantage of current genetic engineering advances to offer an extremely precise way to attack SCLC. BsAbs use offers a novel way to interface the immune system with the tumor to enhance immune mediated tumor destruction. ADCs are revolutionizing the idea of drug delivery specifically to SCLC cells while avoiding the bodies of other cells. Additionally, targeted therapies take advantage of key mutations present in SCLC cells to inhibit cell proliferation and DNA repair, increasing susceptibility to destruction. Research into these various modalities offers patients a vast array of new options in the face of first-line treatment failure. Many of these forms are in their infancy, and research into the efficacy of these modalities and improvements in safety profiles will continue to push the field of SCLC forward and provide efficacious and durable treatments for patients.

## Figures and Tables

**Table 1:** Clinical trials using immune checkpoint inhibitors targeting CTLA-4, PD-1, and PD-L1 to treat SCLC.

Clinical Trial Identifier/Authors	Treatment Modality	Clinical Setting	Treatment Setting	Outcome(s)
NCT02763579/Horn et al. [22]	Atezolizumab plus carboplatin and etoposide	Patients with ES-SCLC with previous treatment failure	1200 mg atezolizumab on day 1 of each 21 day cycle for 4 cycles, followed by atezolizumab until toxicity or progression	Atezolizumab plus chemotherapy achieved a median PFS of 12.3 months compared to 10.9 months in the chemotherapy plus placebo
NCT03043872/Paz-Ares et al. [23]	Durvalumab with or without tremelimumab in combination with EP	Patients with untreated ES-SCLC	1500 mg durvalumab with or without tremelimumab 75 mg EP every 3 weeks for 4 cycles followed by 1500 mg durvalumab	Durvalumab plus chemotherapy achieved a median OS of 13.0 months versus 10.3 months in the chemotherapy group
NCT01331525/Arriola et al. [75]	Ipilimumab plus carboplatin and etoposide	Chemotherapy naïve ES-SCLC	10 mg/kg ipilimumab day 1 of chemotherapy cycles 3 and 6, then once every 12 weeks after week 30	Ipilimumab had an objective response rate of 84.8% and a median immune-related progression free survival of 7.3 months
NCT00527735/Reck et al. [76]	Ipilimumab plus paclitaxel and carboplatin (PC)	Chemotherapy naïve ES-SCLC	Phased or concurrent 10 mg/kg ipilimumab with PC every 3 weeks for 18 weeks, followed by ipilimumab every 12 weeks	Phased ipilimumab had an OS of 12.9 months vs. control of 9.9 months. Concurrent ipilimumab had a median OS of 9.1 months
NCT01450761/Reck et al. [77]	Ipilimumab plus EP	Patients with ES-SCLC	10 mg/kg ipilimumab with EP every three weeks for four cycles	Ipilimumab plus chemotherapy had a median OS of 11.0 months compared to 10.9 months in control group
NCT03703297/ Cheng et al. [80]	Durvalumab plus tremelimumab vs. placebo	LS-SCLC that displayed resistance to chemoradiotherapy	1500 mg durvalumab plus 75 mg tremelimumab or placebo every four weeks for 24 months	Durvalumab plus tremelimumab had a median OS of 55.5 months vs. 33.4 months in placebo
NCT01928394/Ready et al. [81]	Nivolumab monotherapy	LS-SCLC and ES-SCLC with disease progression who failed two or more chemotherapy regimens	3 mg/kg nivolumab every two weeks until disease progression or unacceptable toxicity	Nivolumab monotherapy achieved an 11.9% objective response rate and a median duration of response of 17.9 months
NCT02054806/NCT02628067/Chung et al. [82]	Pembrolizumab monotherapy	Recurrent or metastatic SCLC who failed two or more previous treatment methods	10 mg/kg pembrolizumab every 2 weeks or 200 mg every 3 weeks for up to 2 years	Pembrolizumab monotherapy achieved a 19.3% objective response rate with two patients achieving a complete response
NCT03066778/Rudin et al. [84]	Pembrolizumab plus EP	Stage IV SCLC not previously treated with systemic therapy	200 mg pembrolizumab once every 3 weeks for up to 35 cycles with 4 cycles of EP	12-month PFS estimates in pembrolizumab plus EP was 13.6% vs. 3.1% in control plus EP

**Table 2:** Clinical trials involving the use of PARP inhibitors as monotherapy in SCLC.

Clinical Trial Identifier/Authors	Treatment Modality	Clinical Setting	Treatment Setting	Outcome(s)
NCT01286987/Bono et al. [114]	Talazoparib based monotherapy	Recurrent SCLC patients	Administered 1.0 mg talazoparib daily.	Talazoparib gave SCLC patients with BRCA mutations the highest objective response rate of 40%. 9% of patients had a partial response for 3–4 months.
NCT03009682/Park et al. [115]	Olaparib based monotherapy	Patients who failed prior platinum-based regimen with mutations harboring homologous recombination pathway gene mutation	Olaparib 300 mg twice a day every 12 h administered daily.	Olaparin monotherapy gave an objective response rate of 6.7%. Disease control rate was 26.7%. Median progression-free survival was 1.25 months and median overall survival was 8.56 months.
NCT03516084/Ai et al. [116]	Niraparib based monotherapy	Patients with complete response or partial response to standardized, platinum-based first-line chemotherapy	300 mg niraparib if baseline body weight ≥ 77 kg or platelet count ≥ 150,000/μL. Otherwise, 200 mg niraparib once daily until progression or unacceptable toxicity.	Median progression free survival was 1.54 months. Median overall survival was 9.92 months.

## Data Availability

No new data were created during the drafting of this manuscript.
